# Presentation of breast cancer, help seeking behaviour and experience of patients in their cancer journey in Singapore: a qualitative study

**DOI:** 10.1186/s12885-020-07585-8

**Published:** 2020-11-10

**Authors:** Celene W. Q. Ng, Jennifer N. W. Lim, Jenny Liu, Mikael Hartman

**Affiliations:** 1 Department of Surgery, National University Health System, 1E Kent Ride Road, Tower Block, University Surgical Cluster, Level 8, Singapore City, 119228 Singapore; 2 Institure of Health, Faculty of Education, Health, and Wellbeing, University of Wolverhampton, Wulfruna Street, WV1 1LY Wolverhampton, UK; 3 Saw Swee Hock School of Public Health, National University of Singapore, Block MD6, 16 Medical Drive, Singapore City, 117597 Singapore

**Keywords:** Presentation of breast cancer, Help seeking behaviour, Experience, Singapore, Cancer journey, Qualitative study

## Abstract

**Background:**

Little is known about the presentation, help seeking behaviour for breast cancer in Singapore. Nor was there a study exploring the experience of patients in their breast cancer journey.

**Methods:**

A qualitative interview study with thematic analysis, conducted with 36 patients.

**Results:**

There is no clear pattern of presentation for breast cancer by cancer stage at diagnosis, age and ethnicity in the cancer journey of this group of patients. Patients were diagnosed with early to advanced stages cancer regardless of when they presented or took up treatment in their cancer journey. The reasons patients sought medical attention also did not appear to differ between the stages of cancer diagnosed, ethnic and age. Without setting a measure to define early and late presentation, we found that women shared similar experience in their breast cancer journey, regardless of age, ethnicity and stage of cancer at diagnosis. Poor knowledge of breast cancer (symptoms and causes); few practised regular BSE; denial of symptom; fear of hospitalisation, diagnosis and treatment; worries and stress over financial burden of treatment; misinformation in magazine and online sources; diet; stress; caring responsibility; support network; and use of alternative medicine before and after diagnosis were identified in patients’ narratives. Strong social support; fear of being an emotional and financial burden for the family; and financial worries during treatment were also the recurring themes after diagnosis.

**Conclusion:**

A measure of breast cancer presentation - that accounts for the patient’s experience in the cancer journey, the time interval and tumour biology – that is meaningful to patients, clinicians and researchers is needed. For research on late and delayed presentation, details on BSE practice – how often, when and was it done correctly – will improve the accuracy of time delay interval. For the public, concerted efforts to improve knowledge of breast cancer, survival and prognosis for early-diagnosed cancer, and the importance of regular and correct technique to perform BSE, are critical and urgent to address the rising breast cancer incidence in the country.

**Supplementary Information:**

The online version contains supplementary material available at 10.1186/s12885-020-07585-8.

## Background

Across Southeast Asia, Singapore has the highest incidence rate of breast cancer. Singapore continues to observe a significant increase in breast cancer incidence over the years [1]. Breast cancer is the leading cancer among Singaporean women, accounting for 20% of all female cancers, with an age-adjusted standardised rate (ASR) for mortality of 15.5 per 100,000. While the mortality rate has decreased, the incidence rate has rapidly increased for women over 50 years old. Overall, the incidence of breast cancer is on the rise at a rate of 3%, and the ASR on incidences has increased from 59.8 per 100,000 (2009) to 65.7 per 100,000 (2012). Further, over 22% of advanced stages cancer have been reported: 22.3% (14.4% stage III and 7.9% stage IV) (1995–2007 Singapore-Malaysia Breast Cancer Hospital-based Registry) [2] and 29% (19% stage III and 10% stage IV) according to SEER data for 1975–2013 [3].

Cancer stage at diagnosis is a prognostic factor in breast cancer survival, with an early staging of cancer correlating with improved survival rates [4, 5]. Singapore introduced a national mammography screening programme, Breast Screen Singapore (BSS) in 2002 to improve detection of early stage cancer. BSS invites women aged 50 to 64 years old biennially for screening [6]. Women aged 40–49 and over 64 years have access to BBS upon request. Despite the awareness raising initiatives about mammography screening and breast cancer, improvement of breast care services, and strategies to reduce financial burden, uptake of breast screening remained poor [7]. Only 39.6% attended screening in the previous two years, despite over 90% of women aged between 50 and 69 years being aware of mammography as a breast cancer screening tool. Participation rate was recorded at 9.9% (2002–2003), increased about 4 to 13.7% (2005–2006) but dropped to 11% in 2008–2009. Low uptake of BSS inadvertently meant that symptomatic diagnosis is ever important, but survival is predicated on patients presenting early for symptomatic cancer.

Late or delayed presentation of symptomatic cancer for medical care is a major factor leading to an increase in advanced breast cancer, poor prognosis and higher treatment cost [8, 9]. Patient, healthcare provider, and system-related factors, age, cultural beliefs, attitudes, perception of the symptoms as serious, and anxiety have been reported as factors influencing possible delay in patients seeking health care and treatment [10–16]. In neighbouring country Malaysia, which has the same population ethnic groups (Chinese, Malay and Indian) as in Singapore, late presentation was attributed to the use of alternative medicine, poor knowledge of symptoms, denial, poor social support, missed diagnosis and negative attitudes towards treatment to delayed presentation and diagnosis [17–19]. In Hong Kong, where the population is predominantly Chinese, patients not recognising non-specific atypical symptoms, time factor, barriers to service access, embarrassment and treatment cost were reported [20]. While there are studies investigating the attitudes and low uptake of breast screening among Singaporean women [21, 22], there is yet any study investigating the help-seeking behaviour for breast cancer among patients in Singapore.

Patients’ accounts or narratives have been used to explore and understand patients’ experience of cancer and chronic illnesses since the late 1980s [23–29]. Within qualitative research, narrative and thematic approaches provide systematic means to explore the lived experience and map the richness and wealth of information within narratives. Patients’ account of events and lived experience thus is useful for the purpose of exploring decision making process and action to seek help and treatment. A qualitative approach has been successfully applied to understand the causes and experience of breast cancer [13, 20, 30, 31].

Several definitions have been used to describe late presentation for cancer: delay avoidance [32], time delay [33–36], sources of delay [37], phases of delay [13, 38] and tumour stages [39, 40]. Of these, time delay of 3 months from symptom recognition to medical help seeking is the common definition used to investigate late presentation for breast cancer [18–20, 31, 41], and a shorter time interval of one month has also been used [42]. Apart from the inconsistency in the time interval, there is also concern about the validity of a time delay definition because of the issues of recall bias [43] and the comparability of the results [44]. Breast cancer is a disease that has improved prospects for survival if detected and treated early. Any delay in presentation for symptomatic breast cancer is associated with larger tumours, more advanced stages of disease and consequently poorer prospects for survival [8]. To avoid validity issue due to recall bias, stage at cancer diagnosis has been used as a proxy for time to seek medical care, as it is recognised that those who take longer to seek help are at a greater risk of being diagnosed at advanced stages of cancer [45]. The term ‘advanced stage’ (Stages III and IV) has been used to represent late presentation of breast cancer [4, 46] but this neglects the patient’s perspective of not seeking early medical attention. The role of tumour characteristics and aggressiveness as a prognostic factor in determining the stage of cancer is neglected when focus is placed on patient delay and late presentation [47]. Tumour histology was found to influence the survival of breast cancer patients who delayed hospital admission; with survival greater for cancer of lower grade than higher grade [48]. A valid measure of presentation will need to account for all the above factors.

This paper presents the findings of the first study in Singapore exploring presentation of breast cancer for medical care as experienced by patients in their cancer journey. This study formed part of an international, multicentre qualitative project investigating the psycho-social cultural aspect of breast cancer [49]. Other studies conducted within this project are reported elsewhere [31, 41]. The present paper is an extension to our country comparison study [41], in that presentation and help seeking behaviour for breast cancer by stage of cancer, ethnicity and age; and experience of going through the breast cancer journey in Singapore are discussed in detail.

## Methods

### Sampling design

A nested sampling design, also a form of theoretical sampling aiming to reach maximum variation and heterogeneity within the sample was employed [50, 51]. Our goal was to recruit 54 participants with breast cancer diagnosis from three ethnic groups (Chinese, Malay and Indian) and three age groups (< 40 years, 40–60 years and > 60 years old), with 3 members in each subgroup (Table 1). Previous qualitative studies investigating help seeking for breast cancer reached data saturation between 18 to 46 participants [30].

**Table 1:** Nested sampling design – Singapore study

Ethni-city	Malay (***n*** = 18)						Indian (***n*** = 18)						Chinese (***n*** = 18)					
Breast Cancer Stage	Early Stage Cancer (Stage I & II)			Advanced Stage Cancer (Stage III & IV)			Early Stage Cancer (Stage I & II)			Advanced Stage Cancer (Stage III & IV)			Early Stage Cancer (Stage I & II)			Advanced Stage Cancer (Stage III & IV)		
**Age (years)**	< 40	40–60	> 60	< 40	40–60	> 60	< 40	40–60	> 60	< 40	40–60	> 60	< 40	40–60	> 60	< 40	40–60	> 60
**No. to recruit**	3	3	3	3	3	3	3	3	3	3	3	3	3	3	3	3	3	3

### Recruitment

Ethical approval was obtained from the National Healthcare Group Ethics Review Board, National University Hospital (NUH), Singapore (Reference 2012/02234). Recruitment of patients was conducted at the breast surgery division in NUH where approximately 30% of breast cancer patients in Singapore were managed. Women over the age of 21 years old, diagnosed within the last 6 months with breast cancer (Stages 1 to IV – AJCC 7th Edition Classification,) to minimise recall bias, were identified by their clinical team. They were invited to participate in the study between July to December 2012. To minimise distress, we were careful only to approach the patients after they had chosen their treatment plan and were deemed suitable by their primary oncologist to participate in the study. Apart from the English version patient information sheet (PIS), we also prepared a version in Malay and Chinese languages - patients were provided with the version in their preferred and/or spoken language and a research assistant who was fluent in all the three languages (JLiu) was present to provide explanation about the study. The women were given 24 to 36 h to consider participation before they were contacted by the telephone. A meeting date that coincided with their next clinical visit was scheduled with women who agreed to participate. At the start of the meeting, the research assistant explained the study and obtained verbal informed consent from the participant; this consent was audio-recorded.

### Data collection: interview guide and interviewing process

Data were collected using face-to-face interviews. A topic guide was developed based on previous literature [30], and piloted with 4 patients, and then further refined to local context. The guide covered topics on lay understanding of breast cancer, knowledge and awareness of symptoms and causes, and decisions and behaviour in their journey from symptom discovery to treatment. it followed the patient through the process of symptom discovery, diagnosis, treatment and follow up. Interviews lasted on average of between 30 and 60 min. All interviews were conducted in the patient’s preferred/spoken language in a private clinic room after informed consent was taken. This made the interview process less intimidating, allowing a secure environment for the patient to share their experiences. All the interviews were conducted with the patient; except for five patients (4 elderly Malay patients and 1 Indian patient) who were interviewed in the presence of their children who helped to relay the questions and responses. The research assistant (JLiu) and CN received training on qualitative research from an experienced researcher (JNWLim) for data collection and analysis. All the researchers, except MH were multilingual. A breast care nurse was available for referral if the participant needed psychological advice. Participants were reimbursed for their transportation expenses.

All interviews were audio-taped, transcribed verbatim and, Chinese and Malay transcripts were translated into English during transcription by JLiu who did all the interviews [52]. Interviews were analysed within two weeks before the next interview began. Recruitment stopped after data saturation was achieved.

### Coding and analysis

Two cycles of inductive analysis were conducted. The interviews were transcribed and subjected to applied thematic analysis to determine the behaviour and experience of breast cancer patients in their cancer journey [53–55]. For inter-rater reliability, three researchers in the team (CN, JNWLim and JLiu) read the transcripts independently to identify coding and coded emerging themes into parent nodes and sub-themes. They compared their results and any potential disagreements were identified and resolved following discussion within the team. The emergent themes were then subjected to a second level of analysis for similarities and contrasts by stage of cancer at diagnosis (Stages I to IV), ethnicity and age group. Summary percentages were also calculated to assist interpretation, and to place the findings in the context of the existing literature.

The software package, NVivo (version 10) was used to manage the data. The traditional coding approach of ‘Pen and papers’ was preferred in the second cycle of analysis as this retained the closeness between the researcher and the data.

To ensure robustness of the study, the *Consolidated criteria for reporting qualitative research* (COREQ) was used to plan and implement this study [56].

## Results

Fifty-four patients were approached and 40 (74%) agreed to participate in the study. Only 36 interviews were included in the analysis as four were excluded due to poor recording quality. Of the 36 female patients, 17 were Chinese, 12 were Malay and 7 were Indian; these represented the ethnic distribution of the population in Singapore with 75% Chinese, 14% Malay and 9% Indian. The mean age of the participants was 54 years old (range: 25 to 82 years old) with a majority (81%) aged 40 years old and older. Over a third of the women (13) fall in the BSS programme’s target screening age group of 50–64 years old. Over half of the 36 participants (56%) were diagnosed with early stages cancer while the remaining had advanced stage cancer diagnosis. A quarter of the patients presented with tumour less than 2 cm in size, and about 60% with tumour between 2 cm to 5 cm (Table 2).

**Table 2:** Characteristics of participants

Characteristics of participants		***N*** = 36
**Ethnicity**	Chinese	17
	Malay	12
	Indian	7
**Marital status**	Married	22
	Widowed	2
	Divorced/Separated	0
	Single	7
**Age groups**	< 40 years old	7
	40–49	10
	50–59	5
	60–69	11
	≥70	3
**Stages of cancer at diagnosis**	Early stages	
	Stage 1	9
	Stage II	11
	Advanced stages	
	Stage III	13
	Stage IV	3
**Tumour size (TNM classification)**	T1 (<= 2 cm)	9
	T2 (> 2 cm but < 5 cm)	21
	T3 (> 5 cm, no extension to chest wall)	4
	T4 (any size with extension to chest wall and/or skin)	2

### Presentation of breast cancer symptoms by ethnicity, age and stage of cancer

The most common symptom was a palpable painless lump in the breast regardless of ethnicity, cancer stage at diagnosis and age. Atypical symptoms such as breast pain, tightness and stinging in breast, larger breast size and redness, swelling in breast, boil on breast, and inverted nipple were also reported. There was no difference in the symptoms of breast cancer by age. There were more Malay and Indian women diagnosed with early stage cancer while more Chinese with advanced stage cancer (Table 3).

**Table 3:** Breast cancer symptoms by ethnicity, stage of cancer and age

Ethnicity	Stage of cancer (n)	Stage of cancer	Age group	Symptom(s)
**CHINESE**	**Early stages** (8)	I	< 40	Lump under armpit
		I	40–49	Lump
		I	50–59	Lump
		I	60–69	Lump
		II	< 40	Lump
		II	40–49	Lump
		II	60–69	Lump
		II	60–69	Lump
	**Advanced Stages** (9)	III	< 40	Lump
		III	< 40	Lump, bean size
		III	40–49	Lump, hardness in breast,
		III	50–59	Lump
		III	60–69	Tightness, prickly/stinging pain and later found a lump
		III	60–69	Lump
		III	60–69	Lump (bony)
		IV	60–69	Protruding grape size lump
		IV	< 40	Hard lump, until it grew bigger
**MALAY**	**Early Stages** (7)	I	< 40	Lump
		I	40–49	Lump
		I	40–49	Lump
		II	< 40	Protruding lump on left nipple.
		II	50–59	Lump
		II	50–59	Lump
		II	60–69	A boil of the size of a cherry on the breast
	**Advanced stages** (5)	III	40–49	Protruding lump
		III	40–49	Mammography positive result
		III	50–59	Lump that was painful
		III	60–69	One breast bigger and red.
		III	≥70	Painful lump
INDIAN	**Early Stages** (5)	I	40–49	Had an intuition and went to clinic, Doctor didn’t find lump, she insisted then after 3rd check, lump was found.
		I	60–69	Lump (20 cent coin size)
		II	40–49	Pain in breast
		II	≥70	Swelling lump
		II	≥70	Tiny lump,
	**Advanced Stages** (2)	III	40–49	Pain in breast
		IV	60–69	Inverted nipple, skin ‘pocket’, sometimes got lump

### Presentation by time delay and cancer stage

The data in Table 4 showed that time delay, i.e. interval between symptom discovery and medical help seeking did not reflect the stage of cancer being diagnosed. Seeking medical attention early did not translate into better prognosis. The time delay reported by women diagnosed with early stages cancer (Stages I and II) ranges from immediately seeking medical attention to 6 months. Three women in this group presenting late for medical care from 3 to 6 months and were diagnosed with Stage II cancer. Over half of the women (9/16) who were diagnosed with advanced stage cancer reported delaying medical attention from one month to one year, and one woman said she sought medical attention immediately. Two of the three women with advanced Stage IV said they delayed for 2 months while the other for 7 months. A few women with advanced stage cancer discovered their symptoms after performing breast self-examination (BSE). Regardless of their age, help seeking behaviour among the women appeared to be erratic. These findings suggest that many women did not perform breast self-examination regularly and/or correctly, and discovery of symptoms (painless lump) was opportunistic or by chance.

**Table 4:** Stage of cancer and time delay

Ethnicity	Stage of cancer	Stage of cancer	Age (years old)	Time delay
**CHINESE**	**Early Stages**	I	< 40	1 day
		I	40–49	1 month
		I	50–59	Clinic visit
		I	60–69	1 day
		II	< 40	1 month
		II	40–49	Breast self- examination (BSE), immediate
		II	60–69	6 month
		II	60–69	1 day
	**Advanced stages**	III	< 40	1 week
		III	< 40	1 month
		III	40–49	1 year
		III	50–59	A few days
		III	60–69	7 months
		III	60–69	BSE, immediate
		III	60–69	1 year
		IV	60–69	2 months
		IV	< 40	2 months
**MALAY**	**Early stages**	I	< 40	1 day
		I	40–49	1 month
		I	40–49	Mammogram
		II	< 40	1 day
		II	50–59	3 months
		II	50–59	Medical check up
		II	6069	5 months
	**Advanced stages**	III	40–49	1 year
		III	40–49	Mammography
		III	50–59	5 months
		III	60–69	Hospital admission for other illness/symptom
		III	≥70	1 year
INDIAN	**Early stages**	I	40–49	immediate
		I	60–69	1 month
		II	40–49	1 day
		II	≥70	6 months
		II	≥70	2 months
	**Advanced stages**	III	40–49	A few days
		IV	60–69	7 months

### Patients’ attitudes and experiences in their cancer journey

The narratives of the patients told stories of their attitudes and behaviour in their cancer journey from symptom discovery to treatment.

#### A) Help seeking attitudes and behaviour for breast cancer symptom(s)

Several themes were identified as factors influencing presentation for breast cancer shared by women regardless of their age, ethnicity and stage of cancer diagnosed:

#### Awareness and knowledge of breast cancer

Knowledge of cancer was poor in this group of patients. Participants had neither in-depth nor accurate knowledge of breast cancer prior to their diagnosis, or thinking that cancer is the disease of others. This can be attributed to some extent to ineffective public education but it is apparent that the individual’s disinterest in a matter that they felt will not befall them played an important role.

*‘I only understood it after I was diagnosed with it’* (40–49 year old Chinese female with Stage III cancer).

*‘I have no understanding of cancer as I never thought that I would be diagnosed with it … so I never bothered to understand it (breast cancer).’* (40–49 year old Chinese female with low educational qualifications, Stage II cancer).

*‘Very poisonous. I read the news that when you find out … it will spread. If you don’t seek treatment … it is scary and I don’t dare to think or care about it.’ (*60–69 year old Chinese female, Stage II cancer).

*‘Actually I was shocked also, you know … yeah. Didn’t know it can happen to me also … ’* (50–59 years old Chinese, Stage I cancer).

Knowledge and awareness of breast cancer symptoms and the importance of breast self-examination (BSE) served as barriers to help seeking for medical care.

*‘It’s not life or death, I thought it was natural (to have a breast mass), I was able to do things (and look after the children).’* (50–59 year old Chinese nanny with stage III cancer).

*‘When I felt there was something, because it was on the bone portion, I thought it was more the bone than the lump’* (60–69 year old Chinese female with stage III cancer).

Women who were aware of breast cancer and performed BSE regularly sought medical attention early. This is in contrast to women who delayed seeking help, having poor knowledge of breast cancer symptoms and regarded atypical symptoms as normal and oblivious of its implications.

*‘Normally I don’t massage my own breasts because I don’t have big breasts (it should be obvious if there is a lump)’* (60–69 year old Chinese property agent with stage IV cancer and a 4.5 cm breast lump).

*‘I try to check my breasts every day. That day, I noticed something was inside so I knew there was no need to wait, I went to the polyclinic for a check’* (< 40 year old Malay female with stage I cancer).

*‘ … I raised it (breasts) up to feel, lower it to feel and compare their sizes. Then I realized something was not right, the right one seems to have shrunk and the breasts have become uneven. When you put it down you will know whether they are of the same size. It seems weird today, something is not right. I have not looked at them for two months as I was very busy and suddenly I thought … die. This time I got it’* (60–69 year old Chinese female with stage II cancer).

*‘I did not notice a lump but I had (breast) pain and went to the polyclinic. They told me I had a large lump and sent me to NUH (National University Hospital)’* (40–49 year old Indian female with stage III cancer).

*‘I know how to check, but I never checked (my breasts)*’ (40–49 year old Indian female with stage III cancer).

#### Misinformation

Sources of public education such as mass media, magazines, search engines and television shows can act as an enabler if accessible to the right population groups. Likewise, they can also be barriers if inaccurately presented.

*‘I learnt how to examine my breasts from a television program. I thought that if I had a lump, the shape will definitely change … and this is true’* (60–69 year old Chinese with Stage II cancer).

*‘I went online and searched for some information. It said that if it could move, it might be benign.’* (< 40 year old Chinese graduate with Stage III cancer).

*‘One day I was reading a magazine about this Malay lady being diagnosed with cancer and the symptoms of it. One of them was having a lump beneath the armpits. Then I happen to have a lump beneath my armpits … Singapore had this Pink Ribbon event organized by the Breast Cancer Awareness Society, giving us some information’* (< 40 year old Chinese graduate with Stage I cancer).

It is a common norm to seek the opinions of family members and loved ones, and then draw conclusions based on their knowledge and experiences.

*My mother had a benign lump before, she did not remove it and it recovered on its own. So she told me that mine might be the same. I trust my mother’s words so I did not seek treatment immediately. About a week later, I pondered about it and felt the lump* (< 40 year old Chinese graduate with Stage III cancer).

#### Diet and stress

Diet and stress were thought to be major contributors of cancer and general prevention of ill health by diet modification and good exercise regimes were thought to prevent cancer.

*‘When you talk about causes (of breast cancer) … I have heard that I have to eat healthy … for 10 years I did not eat McDonalds, or any fried food, I don’t even know how to order food at those restaurants … ’* (60–69 year old Chinese with stage III cancer).

*‘ … Sometimes what we eat, consume, plays a part’* (50–59 year old Malay retail assistant with recurrent stage II cancer).

*‘What causes it? Could be stress, could be your food. I mean those kinds of things and many different factors’* (40–49 year old Chinese Librarian with stage I cancer).

#### Family history of breast cancer

While most knew that genetics played a role in predisposition of cancer, a negative family history gave them false reassurance.

*‘What we understand is that cancer is related to (family) history and genes right? So … so that’s why everybody just ignored, and never went for check-up … because we believed that we were safe since our family does not have any history of this (breast cancer)’.* (50–59 year old Malay female with recurrent stage II cancer).

*‘I didn’t even know that one could contract this illness if there was no history of cancer in the family’* (40–49 year old Chinese female with low educational qualifications and stage II cancer).

*‘The weird thing is, none of them (parents) have this, they have diabetes, I don’t understand why I got this’* (60–69 year old Chinese administrator with Stage III cancer).

#### Fatality of breast cancer

Some believed that breast cancer was fatal and that the treatment was futile.

*‘Feels like once you get it, you will die from it’* (< 40 year old Chinese unemployed with stage II cancer).

*‘ … But this sort of illness, to me, this kind of illness is very scary. Even in its early stages, it is difficult to be cured.’* (60–69 year old Chinese accountant with stage II cancer).

*‘My understanding is it could be fatal, it means it could be quite life threatening. It is treatable, but it is not, there is no warranty of cure once you have cancer, any kind of cancer, inclusive of my breast cancer.’* (60–69 year old Chinese property agent with stage IV cancer).

#### Breast cancer screening

A third of the patients displayed awareness of breast screening. However, this did not translate into productive actions due to perceived pain associated with mammograms.

*‘I did not want to go for mammograms because it is painful’* (50–59 year old Chinese nanny with stage III cancer).

#### Caring duties

Women reported delaying seeking medical attention because of caring duties. Some of this could be attributed to the cultural fixation on multiple roles of the female – a mother, wife, daughter, provider, and friend.

*‘I did not dare tell my family. I tell myself not to bother (them). All the chemotherapy and radiotherapy are troublesome and I would still die after (the treatment)’* (60–69 year old Chinese female with stage I cancer).

*‘I knew about it last year, but I kept quiet. I have to look after my husband – his cataracts (before I go and see the doctor)’* (50–59 year old Malay housewife with stage III cancer).

*‘If I went for surgery (5 years ago when I was still a nanny), nobody will look after the child, things would be difficult for the mother.’* (50–59 year old Chinese nanny with stage III cancer).

#### Fear

Fear was a recurring theme. It is an irrational emotion that encouraged productive and counter-productive behavior. Women were often stunted by fear and hid behind purposeful oblivious leading to delayed presentation.

*‘I realized there was something wrong (about 6 months ago), but I did not check up immediately, I dragged it out for about 3 to 4 months … I was afraid, what if it is a malignant tumour? Actually I always hear from others that it should be during the early stages when you are detected … but this sort of illness, to me, this kind of illness is very scary.’* (60–69 year old Chinese accountant with stage II cancer).

*‘I know that I got (cancer), and I have to go to the hospital. I was already scared, so I postponed to see doctor, I delayed and delayed, and then I realized … I don’t know where to go, I cannot find out how to cure this sickness, so finally I come here (hospital)’* (40–49 year old Malay female with stage III cancer).

*‘ … knew it could be cancer, tried to dismiss the idea away because all her life she has never been to the hospital’* (60–69 year old Malay female with stage III cancer).

#### Patient’s personality

Inherent personality traits were important factors that could override barriers to seeking medical attention. Resilient and analytical personalities were evident in some women who presented early. Though fear was unanimously encountered, they were able to overcome the situation and made the sensible decision to seek help early.

*‘On one hand I was very scared I would die … but I thought that the most important thing would be to heal myself first’* (40–49 year old Chinese female with low educational qualifications and stage II cancer).

*‘I panicked, thinking if this could be cancer … I tried to remain calm and immediately went to the clinic’* (60–69 year old well-read Chinese female with stage III cancer).

*‘After you find a (breast) lump, be it good or bad, you should accept it and seek treatment immediately’* (60–69 year old Chinese accountant with stage II cancer).

#### Fear of dying in the hospital

Two elderly Malay patients conceived their symptom from their family because they believed that they would die lonely in the hospital.

*‘ … I would die there (hospital). I could tell my son, I kept this secret until it was too painful … ’ (*over 70 year old, Malay, Stage III cancer).

*‘I might die in the hospital if I go there’* (over 70 year old, Malay, Stage II cancer).

#### Traditional and alternative medicine

The easy accessibility of traditional and alternative medicine locally is a double-edged sword. Patients often choose to subscribe to alternative medicine believing in its curative effect, thus delaying presentation.

*‘I decided to wait and see, I was doing some alternative treatments, diet, exercise and you know, good healthy lifestyle. I stopped taking sweets and meat … ’* (60–69 year old Indian Housewife with stage IV cancer).

*‘I went to the traditional Chinese medicine doctor first (after being diagnosed at the polyclinic)’.*

(< 40 year old Chinese graduate with stage IV cancer)

*‘ … I’m now looking for a traditional and alternative medicine to improve my health, as there are many side effects after chemotherapy, like cough. After my second chemotherapy, I had a cough and it did not recover for two months. After that I went to see a TCM until now.’* (< 40 year old, Chinese, Stage III cancer).

#### Financial burden

Healthcare in Singapore is a fee for service system. A few patients decided to wait because they were worried about the financial burden placed on themselves and their family. One waited until she was offered a job.

*I really did not want to see the doctor when I found the lump. The treatment cost is expensive in Singapore. I have Medisave and my son also has saving. But it would be a lot of money. I waited and hope it would go away. When I finally had treatment, they told me I am qualified for government subsidy, I have no knowledge of this subsidy. Now I am in the clinical trial*. (60–69 year old, Chinese, Stage II cancer).

*‘I thought of seeing the family doctor but that would be expensive. How much is one (referral) letter? Better not. Let’s go poly (clinic).’* (60–69 year old, Chinese, Stage II cancer).

*‘If you fail your medical check-up, they (the employer) will terminate you, so I wanted to check after I got the job’* (50–59 year old, Malay, Stage II cancer).

#### Family support

Family members acted as the motivating factor for women to seek help for their symptom(s).

*‘Initially I told my children not to bother about it, but later I thought about it and I think it is better to see a doctor as my grandchildren are so young and cute, and I cannot bear to leave them. She (my daughter) said “Mum, I will take the day off and bring you”.’* (60–69 years old, Chinese, Stage I cancer).

*‘I had the symptom, a lump, for a while. I found it while I was holidaying in Indian. Then I visited my son in the US and told him. He scolded me and told me to see the doctor immediately. I came back to Singapore and went to the doctor’* (60–69 year old, Indian, Stage IV cancer).

#### B) Patients’ experience after diagnosis and during treatment.

Women told their experience after they were diagnosed with breast cancer, and the coping strategies they adopted during treatment.

#### Acceptance of cancer

Older patients expressed acceptance of diagnosis and fate, while a young patient expressed shocked.

*‘There’s nothing much. If I have to face the later stages of (cancer) that cannot be treated and will lead to death, there’s nothing we can grumble about. To put it plainly, if your time is up … you have to take care of yourself. Such problems cannot be prevented.’* (60–69 year old, Chinese, Stage I cancer).

*‘ … I was struggling the past few months, therefore I was mentally prepared when the doctor confirmed the diagnosis. I was mentally prepared before visiting the polyclinic and accept … hence when I knew the truth I just accepted it.’* (60–69 year old, Chinese, Stage II cancer).

*‘My children cried and I asked what’s there to cry about? I said people live and die. I was calm when the doctor told me (about the diagnosis) too.’* (60–69 year old, Chinese, Stage III cancer).

*‘On the day of surgery, when I was on the surgery operating theatre’s bed, I cried because I was thinking why I am so unlucky that this is happening to me. I’m not afraid of death or what … just sighing that I am so unlucky to have this*.’ (60–69 year old, Chinese, Stage I cancer).

*‘I was shocked. After that I broke down and cried. I couldn’t accept it.*’ (< 40 year old, Chinese, Stage III cancer).

#### Anxiety over treatment cost

Some women worried about financial burden while undergoing treatment.

*‘A clinic visit costs over a hundred over dollars every time... Consultation costs about $30, scans are at least a hundred dollars, what about the weekly blood tests if you go on chemotherapy. You have to pay by cash. Therefore people say Singaporeans can die but not fall sick.’* (60–69 year old, Chinese, Stage III cancer).

*‘I don’t want to burden my family. … my parents were very sickly … I have eight siblings and we couldn’t afford their medical bills. We were not educated and we earn about $1-2000 monthly, how is that even enough to support our own family. … after my parents passed on, its like a piece of shattered glass … I have seen my parents’ condition and I don’t want to burden my children. It is a hundred over dollars every time. You can imagine how much it costs when I visit these three places, What about weekly blood test? It costs $30 every time I see the doctor..’* (40–49 year old, Malay, Stage I cancer).

#### Indecision about post-surgery care

One patient described the process she went through to decide if she should have a breast implant or tissue grafting after her mastectomy.

*‘Day one, you want. Day two, you don’t want. Day three, you want. It’s a lot of back and forth and a lot of opinions from people that influence you to see whether you should. … Why do another surgery to get … your breast back. … started looking the health effects, the cost, the long term effects. Especially if let’s say, the surgeon did suggest that I do an implant, rather than a tissue grafting. Then you start looking on internet and check, whether it is okay and the long term effects. After weighing all the health … potential health risk, potential financial cost, and all these things. I felt that it would, I’m not keen on the tissue grafting, and I feel like it is two major surgeries. I already have one big one (surgery), I don’t want another one, so that is sort of out. So I’m left with either implant or with don’t have any reconstruction. Implant was one of the potential problems that comes with it, it’s going to last ten, fifteen year, but who knows, it may not last ten or longer or maybe shorter then you have more problem. So looking at the cost (and) financial problems, what I want to do from now on, so I felt that maybe it’s not necessary after all. Although at one stage, I felt that I need it’* (40–49 year old, Chinese, Stage I cancer).

#### Diet and stress

Women talked about their acceptance that diet and stress caused their cancer and made changes to the lifestyle.

*‘More concerned about whether any kinds of food could be the cause … I gave up seafood except fish … I still eat chicken because I believe chicken is white meat, … I changed my eating habits by taking more fruits. I am also of the opinion that cancer patients like me are not disciplined. I think it’s about discipline with food.’* (60–69 year old, Chinese, Stage IV cancer).

*‘But now I understand that cancer could be due to stress, the environment and food’* (< 40 year old, Chinese,Stage III cancer).

*‘Even now with the relapse, it could also be due to stress, due to my work, like last time. So now I just want to think less and not put too much pressure on myself’* (50–59 year old, Malay,Stage II recurrent breast cancer).

#### Traditional and complementary medicine

One patient reported turning to traditional and complementary medicine to cure the cancer after diagnosis. Another used traditional and complementary medicine to cope with the side effects of treatment.

*‘I went to the doctor at the polyclinic and was diagnosed with the cancer, then I went to see a traditional healer. He said he will heal me. After 1 month, nothing happened, I came to the hospital and they said I have to do chemotherapy … ’* (< 40 year old, Chinese, Stage IV cancer).

*‘Actually I’m now looking for a TCM to improve my health, as there are many side effects after chemotherapy, like cough. After my second chemotherapy, I had a cough and it did not recover for two months. After that I went to see a TCM until now.’* (< 40 year old, Chinese, Stage III cancer).

#### Recalling the efficiency of the breast cancer healthcare and referral system

While one patient complained about her expectation to be recalled by the hospital because she did not follow up or missed her specialist appointment after her mammography, others have praised the efficiency of the breast health structure and referral system.

*‘… Sometimes the patient might not follow up actively. But the hospital should have some form of follow up, like sending a letter to advise the patient to return to the hospital for a check-up and not wait for a normal check up every two years.* (60–69 year old, Chinese, Stage II cancer).

Many patients appreciated the convenient and efficiency of the breast health system in serving their needs.

*‘When I was about to go home, they called to inform me that there is an available slot on the same day, So I went to the hospital immediately and did all the necessary procedures.’* (60–69 year old, Chinese, Stage III cancer).

*‘I went to a clinic and the (doctor) told me it is (cancer). He helped me make a call immediately, he called up NUH and he said I could have (an appointment) in a week.’* (60–69 year old, Chinese, Stage III cancer).

*‘Polyclinic is the best, they do a check-up for you and look for the relevant doctors … he immediately called the (specialist) hospital when he felt something was wrong … getting an appointment through them is very fast and cheap … you know a family doctor will take 2 to 3 weeks and would be expensive. I saw the doctor at 11 am that day and he made me an appointment at 3 pm on the same day’ (*60–69 year old, Chinese, Stage II cancer).

*‘I called to make an appointment for the scan (mammogram), but the staff asked me if I had a lump, and recommended me to go to see the doctor immediately instead’* (60–69 year old, Chinese, Stage III cancer).

#### Prayers to god

The majority of Singaporeans are religious. Thus, it’s natural that some patients turned to God to ask for recovery and miracles through the doctors.

*‘From my viewpoint of as Christian, god can still heal through medical means, through doctors or whatever. He can always do it, like using healing, that sort of things’* (40–49 year old, Chinese, Stage I cancer).

*‘Well, of course prayer, prayer has always been part of my life. So I believe God heals. But I think at the same time, God provides the doctors and I think God created all plants and animals for food. So I believe plant based there are many extracts used in medicine these days. I think Soursop is one of the known plants’* (40–49 year old, Indian, Stage I cancer).

#### Support network

A central theme that transcended all sub-themes and re-surfaced repeatedly was support network. It was able to break down barriers that stemmed from both emotional and structural origins. Family involvement was different for each patient but the presence of family in itself was a universally enabling factor for seeking specialist evaluation after a diagnosis.

*‘I don’t know what to do; my sister replied everything for me. She did everything for me; I didn’t know what to do’*. (< 40 years old, Chinese, Stage IV cancer).

‘*We went home and discussed this (treatment) method with our children, after all our children already grown up, so we just discussed. I did not want to wait any more longer, so we decided to go on with the surgery because it’s only at the early stage. We don’t need to let it worsen, so that’s why as early as possible, we get it out.’* (40–49 year old, Malay, Stage I cancer).

*‘Even though I am a widow, I have both my children, they are very supportive, I went (to the specialist) with my daughter’* (Over 70 year old, Indian, Stage II cancer).

*‘My husband was with me at the point where they talked about the results from the biopsy, so he was the first person to know. He cried.’* (40–49 year old, Indian, Stage I cancer).

*‘My friend was present when I received my diagnosis. We cried together. I went home and told my family. My mother and the rest were sad, my father cried as well. They don’t really believe it and asked me to do another test.’* (< 40 year old, Chinese, Stage III cancer).

## Discussion

Our study is the first and only qualitative study specifically exploring the help seeking behaviour and presentation of breast cancer among patients in Singapore. In this paper, we were interested in the help seeking behaviour of patients regardless of the time they took to seek medical attention after symptom discovery, and compare this by stage of cancer, age and ethnicity. Because tumour biology and aggressiveness is a prognostic factor for stage of cancer at diagnosis, we purposefully did not use any measure to categorise the patients into early or late presenters. We found no pattern in the presentation of symptoms by age and ethnic group, neither is there a positive relationship between stage of cancer at diagnosis and time delay (interval between symptom discovery and first medical help seeking). Presentation of cancer also did not appear to differ between the stages of cancer diagnosed, ethnic and age groups. These findings suggest that a need for a definition or measure of late/delayed presentation that is meaningful for patients, clinicians and researchers [44] – one that accounts not only for time interval, the cancer journey as experienced by the patients, and the tumour biology.

We observed that women who presented early (less than 3 months) or immediately were also diagnosed with advanced stage cancer (Stages III and IV), and vice versa. Apart from tumour biology which affects the cancer staging at diagnosis [48], this finding could be explained by chance discovery, opportunistic health check and mammography. The women would have no knowledge that they had a palpable lump unless they regularly and correctly performed BSE, or had a health check and mammography. Based on our clinical experience, a lump of 2 cm is usually palpable by the woman and the doctor; since 75% of this patient group had tumour over 2 cm, it can be assumed that these patients did not perform regular BSE or correctly performing the techniques, and discovered their symptom either by chance, or through opportunistic health check and mammography and sought medical advice, or they ignored the symptom and delayed help seeking.

The length of time delay is assessed by the patient directly and although there is concern about recall bias, it has been observed that women were careful to record the time when they first noticed their breast cancer symptoms [43]. However, for an accurate time delay, researchers would need to further establish if BSE was performed regularly and correctly. Without this information, validity of the time delay information is questionable, as the information provided by the women did not capture the exact period of delay when the lump became palpable or atypical symptom manifested. Time delay interval from symptom discovery to help seeking is not an accurate measurement for patient delay unless further information on regular and correct BSE practice are collected. For this group of women, it would appear that BSE was not a common practice. Further, time delay measure accounts only the time period between symptom discovery and first medical consultation, and any delay after diagnosis is not considered.

A third of the women in this patient group fell into the BSS target screening age group and none reported taking up the biennial invitation. Uptake of BSS mammography screening in Singapore has been low; 90% of women aged 50–69 years are aware of mammography as a screening tool but less than 40% had ever taken up a test [7]. Another study reported that cancer knowledge predicts uptake - 41% of eligible women with limited cancer knowledge compared to 68% with high cancer knowledge had ever been for mammography screening (*p = 0.01*) [22].

Without setting a measure to define early and late presentation, we found that women shared similar experience affecting their breast cancer journey, regardless of age, ethnicity and stage of cancer at diagnosis, in their cancer journey. They reported poor knowledge of breast cancer (symptoms and causes); few practised regular BSE; denial of symptom; fear of hospitalisation, diagnosis and treatment; worried and stress over financial burden of treatment; misinformation in magazine and online sources; diet; stress; caring responsibility; support network; and use of alternative medicine before and after diagnosis. Among these, strong social support; fear of being an emotional and financial burden to the family, and financial worries during treatment were recurring themes. These findings are similar to studies that used a time delay of 3 month and over to define delayed or late presentation [18–20, 31, 41].

Existing evidence within Singapore suggested that knowledge, educational level, patient perception of cancer and the efficacy of a screening program constitute barriers to early presentation of breast cancer [22, 57, 58]. Our study concurred that a major barrier to presentation that was consistent throughout the three groups and across all age groups, was the lack of, and inaccurate knowledge of cancer and its causes. However, we found that this was evident even in younger patients with professional degrees. This could be due to the inherent disinterest in a disease that the patients’ perceived as unlikely to befall them or the disease of others. Patients also equated cancer to death sentences resulting in fear and an escapist mindset leading to delayed presentation.

A unique theme was the unwavering sense of personal responsibility to the family and their careers. Women had prioritized work and their family above their own health, fiercely refusing to be a burden either emotionally and financially to their family. This phenomenon is likely borne from the influence of the Asian culture and universal expectations of women in their multiple roles as a wife, mother, daughter and income provider. Further, many patients emphasised the importance of family support in getting the care they need in their cancer journey.

The global shift toward a healthy living culture with meditation, health foods and exercise invariably popularizes alternative medicine. Though it has a role in specific diseases, patients often mistake it as effective cancer treatment thus delaying their presentation. A positive impact of this trend is the heightened interest in BSE. Women diagnosed with early stage breast cancer were able to pick up abnormalities prompting them to seek help early. While those with late stage disease either did not examine their breasts altogether or did so with poor technique and missed even large lesions. This emphasizes the need for education about BSE and skills to perform the technique correctly.

Singapore practices a co-payment healthcare scheme urging citizens to participate actively in their healthcare. As a healthcare hub, specialist centers such as the breast center in National University Hospital, has triple assessment housed within one unit. Clinical staffs are trained to assist patients in their breast cancer journey, providing psychosocial and wound care services. Primary healthcare practitioners have direct access to clinic appointment slots in order to facilitate a prompt and seamless workup and treatment. The convenient and efficiency of the health care system for breast cancer referral from primary to secondary care was an enabling factor for many patients. Patients reported visiting the polyclinic for breast cancer symptom as their first health access point, and then, immediately referred to the specialised breast clinic in secondary care for diagnosis. They have praised these features, and the expertise of their healthcare professionals. However, as a fee-for service health system, the financial burden of the disease had resulted in patients delaying help seeking, and for some patients, had caused worries about their treatment cost.

Our study is not without limitations. We recruited patients through their clinical team. This method could have caused undue influence whereby patients might feel obliged and agreed to participate in the study because they were asked by the clinician or that they were indebted to the care they received. We did provide 24 to 48 h to the patients to consider participation and at the start of the first meeting, while collecting informed consent, we did assure patients that their standard of care would not be violated should they decide not to participate or withdraw while in the midst of the study. Another limitation of our study is that we did not apply a measure to define early and late presentation and thus, restricts comparison with studies that used time delay or stage of cancer as measure. However, our approach provided another perspective to the analysis. We were able to allude that other factors such as tumour characteristics and aggressiveness are relevant in studying presentation and help seeking behaviour. Further, we showed that time delay is not an accurate measure if information of regular and correct technique to practice BSE are not elicited.

## Conclusion

There is no clear pattern of presentation for breast cancer by cancer stage at diagnosis, age and ethnicity in this group of patients in Singapore. Regardless of when they presented and/or took up treatment in their cancer journey, patients could still be diagnosed with advanced stage cancer. Tumour biology plays an important role in staging of cancer diagnosed, as well as whether women practice regular and correct BSE technique. A measure of breast cancer presentation - that accounts for the patient’s experience in their cancer journey, the time interval and tumour biology – that is meaningful for patients, clinicians and researchers is needed. For research on late and delayed presentation, details on BSE practice – how often, when and was it done correctly – will improve the accuracy of time delay interval. For the public, concerted efforts are urgent and critical to improve the knowledge of breast cancer, survival and prognosis for early-diagnosed cancer, the importance of regular and correct technique to perform BSE, and to allay financial worries of the disease in Singapore.

## Supplementary Information

**Additional file 1.**

## Data Availability

Not applicable.

## Abbreviations

NUH: National University Hospital, Singapore
BSS: Breast screen Singapore
AJCC: American Joint Committee on Cancer
BSE: Breast Self-examination
