# Supplementary material for: Presentation of breast cancer, help seeking behaviour and experience of patients in their cancer journey in Singapore: a qualitative study
Source: BMC Cancer. 2020 Nov 10;20:1080. doi: 10.1186/s12885-020-07585-8 (PMC7653763; doi:10.1186/s12885-020-07585-8)
Supplement: Supplementary file 1 — Additional file 1. [file 12885_2020_7585_MOESM1_ESM.pdf]

# INTERVIEW GUIDE – Singapore_Presentation of breast cancer_2012

**(permission of use to be obtained from the authors)**

**KEY THEMES TO EXPLORE in details**
**(no particular order – follow flow of interviewees)**

- Help seeking process (if possible, to determine the timeline in the right order of incidences)
- Symptoms identification/recognition
- Coping strategies (denial/avoidance, lay referral network, social support and network/ alternate or traditional medicine)
- Lay understanding of disease
- Fatalism, stigmatisation

**<u>FOLLOW THE TIMELINE</u> from the first symptoms to the first contact of medical professionals and official diagnosis. <u>FOLLOW THE NATURAL FLOW OF THE STORY AS TOLD BY THE PATIENT AND ASK QUESTIONS AS APPROPRIATE.</u> Use patient <u>APPROPRIATE LANGUAGE AND CONCEPTS</u>; if a patient does not understand eg "symptom" use expressions as "health problems" or similar:**

***Identifying help seeking process and symptom(s)***

1. When did you first feel/think that something might be wrong with your health? **(warm up question, also exploring the beginnings of the health problem)** Or When were you first alerted that something was wrong with you? **(if a person was alerted by somebody else, eg screening or doctor's examination)**
2. Do you believe in screening? **(if screening can help to detect health problem)**
3. What kind of symptom(s) did you have? Why did you feel/think something was wrong? **(first symptoms, illness recognition)**
4. How did this (these) make you feel/ think? **(impact of symptoms on life at a particular moment)**
5. How long did you take to consult the doctor for check up from the time you thought/felt that something was wrong with your breast? **(days/months – if patients can provide an approximate answer)**

***Coping strategies (to identify and explore negative and positive)***

6. What did you do about them? What helped you? What did not help you? **(coping strategies – patients may refer to denial and avoidance; patients may refer to alternative medicine/lay referral and social networks/etc)**
7. Possible probing questions for **denial/avoidance**: what were you afraid of? What did you hope to happen by avoiding the problem? What and how long did it take for you to accept that there was a problem?
8. Possible probing questions for **lay referral and social support networks**: Who did you talk to? What type of help did you get from this person? What type of advice did you get? How did this person help you (transportation/interpreter/emotional support/company)?
9. When you first mention this symptom(s) to your significant other (spouse/family members), what was his or their advice?

10. Further probing questions: How (if at all) did other people help you/ didn't help you? Who helped you with what **(eg. recommending to go to see traditional healer, opined there is nothing to worry about etc)**
11. **If not mentioned:** Have you at any point consulted **traditional/ alternative/ religious healer**?
12. When you visited the doctor, what were you hoping to find out from the doctor? Did you think that medical treatment can cure your disease?
13. What happened next?

**Follow and repeat questions as appropriate, the narrative ends with official diagnosis.**

***Lay understanding of disease***

14. How would you explain to another person what your disease is? Is this ('cancer') a disease you often heard being talked about? **(lay understanding of cancer)**
15. **Be careful, do not personalise this question because patients might feel personally responsible for getting cancer anyway – if you suspect this do not ask this question, but explore if the patient raises it herself )** What do you think causes breast cancer? **(lay aetiology of cancer)** Do you think it is contagious?
16. Which symptoms /problems ( eg not being able to take care of the children properly anymore) were the most difficult? Why? How have you dealt with them? Which symptoms (problems) were the least difficult? Do you think anything positive happened because of your illness **(illness related benefit)?**
17. What would you do differently if you knew what you know now? What would you recommend to other women?

***Fatalism, stigmatisation of disease in the family and community***

18. What do you think of when you heard of the word 'cancer'? (**Explore patients' perception and beliefs about cancer when patients first thought of the disease)**
19. Did anyone in the family have this disease? In the community? Is this an openly talked about subject?
20. Do you think by talking about 'cancer', this can bring bad luck to you or/and your family?

***Mapping help-seeking process***

**Ask patient to determine her help-seeking process on a timeline (this may not be a straightforward or continuous process). Identifying what happened next. Patients may refer to traditional/alternate medicine after they visited the doctor.**

1.symptom recognition → 2.____ → 3.____ → 4.___ →5._so on___ →6. Visit the doctor →7. ____
